# Independent confirmation of juvenile idiopathic arthritis genetic risk loci previously identified by immunochip array analysis

**DOI:** 10.1186/1546-0096-12-53

**Published:** 2014-12-16

**Authors:** Rachel C Chiaroni-Clarke, Jane E Munro, Raul A Chavez, Angela Pezic, Roger C Allen, Jonathan D Akikusa, Susan E Piper, Richard Saffery, Anne-Louise Ponsonby, Justine A Ellis

**Affiliations:** Genes, Environment & Complex Disease, Murdoch Childrens Research Institute, 50 Flemington Rd, Parkville, Victoria 3052 Australia; Department of Paediatrics, The University of Melbourne, Parkville, Vic 3010 Australia; Arthritis & Rheumatology, Murdoch Childrens Research Institute, 50 Flemington Rd, Parkville, Victoria 3052 Australia; Paediatric Rheumatology Unit, Royal Children’s Hospital, 50 Flemington Road, Parkville, Victoria 3052 Australia; Environmental and Genetic Epidemiology Research, Murdoch Childrens Research Institute, 50 Flemington Rd, Parkville, Victoria 3052 Australia; Paediatric Rheumatology Department, Monash Children’s Hospital, Monash Medical Centre, 246 Clayton Road, Clayton, Vic 3168 Australia; Cancer & Disease Epigenetics, Murdoch Childrens Research Institute, 50 Flemington Rd, Parkville, Victoria 3052 Australia

**Keywords:** Juvenile idiopathic arthritis, Immunochip, Independent replication, Genetic association

## Abstract

**Background:**

Our understanding of the genetic factors underlying juvenile idiopathic arthritis (JIA) is growing, but remains incomplete. Recently, a number of novel genetic loci were reported to be associated with JIA at (or near) genome-wide significance in a large case–control discovery sample using the Immunochip genotyping array. However, independent replication of findings has yet to be performed. We therefore attempted to replicate these newly identified loci in the Australian CLARITY JIA case–control sample.

**Findings:**

Genotyping was successfully performed on a total of 404 JIA cases (mean age 6.4 years, 68% female) and 676 healthy child controls (mean age 7.1 years, 42% female) across 19 SNPs previously associated with JIA. We replicated a significant association (p < 0.05, odds ratio (OR) in a direction consistent with the previous report) for seven loci, six replicated for the first time - *C5orf56-IRF1* (rs4705862), *ERAP2-LNPEP* (rs27290), *PRR5L* (rs4755450), *RUNX1* (rs9979383), *RUNX3* (rs4648881), and *UBE2L3* (rs2266959).

**Conclusions:**

We have carried out the first independent replication of association for six genes implicated in JIA susceptibility. Our data significantly strengthens the evidence that these loci harbor true disease associated variants. Thus, this study makes an important contribution to the growing body of international data that is revealing the genetic risk landscape of JIA.

**Electronic supplementary material:**

The online version of this article (doi:10.1186/1546-0096-12-53) contains supplementary material, which is available to authorized users.

## Findings

Juvenile Idiopathic Arthritis (JIA) is the most common rheumatic disease in children [1], yet there are relatively few confirmed disease associated loci, and we are therefore limited in our understanding of the overall genetic architecture [2, 3]. Only two genome-wide association studies have been published for JIA [4, 5], limited by sample size and/or genomic coverage. In these studies, a discovery sample was used to identify potential risk loci, and then an independent replication sample was used to confirm the findings. In both studies, a number of loci were identified as associated with JIA in the discovery sample that were unable to be replicated in the validation sample [4, 5]. This highlights the importance of independent replication in determining true disease associated loci.

In 2013, Hinks et al. published the most successful genetic association study for JIA to date [6], using the Immunochip [7]; a genotyping array specifically designed to densely capture variation at immune-related genetic loci. The study further validated the well-established associations of HLA, *PTPN22* and *PTPN2* with JIA, in addition to confirming the involvement of other loci such as *STAT4* with JIA, that were previously associated with the disease, but not at a genome-wide level of significance (p < 5 × 10^-8^)[6]. More notably, the same study identified a suite of novel JIA risk loci reaching either genome-wide significance, or a genome-wide ‘suggestive’ level of significance (p < 1 × 10^-6^) [6]. However, in order to maximize statistical power for discovery, all available JIA cases formed the discovery sample, and no replication sample was available to confirm these novel associations. Therefore, we aimed to confirm the involvement of these new JIA risk loci in an independent sample of JIA cases and healthy child controls drawn from the CLARITY JIA case–control study in Melbourne Australia [8, 9].

We selected 22 single nucleotide polymorphisms (SNPs) from the JIA Immunochip association study [6], for potential replication. SNPs that are established susceptibility loci for JIA [4], or that have previously been associated in our sample [8] (either the SNP itself or a SNP in high linkage disequilibrium) were omitted. Two SNPs selected for inclusion subsequently failed assay design and were therefore also excluded. Reasons for exclusion are listed in Additional file 1: Table S1. Genotyping of the remaining 20 SNPs was attempted in a total of 408 JIA cases and 687 controls drawn from CLARITY, using the Sequenom MassARRAY system (assay design details available from the authors). Following exclusion of individuals and SNPs not meeting quality control thresholds (<90% call rate for individuals or SNPs, and departure from Hardy Weinberg equilibrium p < 0.001), genotype data was successfully generated for 404 JIA cases (mean age 6.4 years, 68% female) and 676 healthy child controls (mean age 7.1 years, 42% female) across 19 SNPs (listed in Table 1). One SNP, rs66718203 at *PRM1-RM12*, failed genotyping quality control and was excluded from further analysis. We re-genotyped a random 10% selection of samples across all SNPs and achieved a genotype concordance rate of >99%. Allelic, genotypic, additive (Cochrane-Armitage test for trend), dominant and recessive association tests were performed using PLINK v1.07 [10]. We considered p < 0.05 for any test, along with an allelic odds ratio (OR) in a direction consistent with the previous report, as evidence of replication. As these SNPs had *a priori* evidence for association, we did not adjust the p values for multiple testing.

**Table 1:** **CLARITY association replication results**

Gene	SNP	Minor allele	Case MAF/Control MAF		Allelic	Best	Immunochip		Replication ^c^
				P	OR (95% CI)	P ^a^	P ^b^	OR (95% CI)	
*13q14*	rs34132030	T	0.30/0.30	0.82	1.02 (0.85-1.24)	0.68 D	1.77 × 10^-7^ A	1.18(1.11-1.26)	N
*AFF3-LONRF2*	rs6740838	T	0.44/0.41	0.25	1.11 (0.93-1.32)	0.042 R	8.83 × 10^-7^ D	1.25(1.14-1.37)	Y
***ANKRD55***	rs71624119	A	0.20/0.23	0.10	0.83 (0.67-1.03)	0.081 R	4.40 × 10^-11^ A	0.78(0.73-0.84)	S
***ATP8B2-IL6R***	rs11265608	A	0.08/0.07	0.50	1.12 (0.81-1.55)	0.50 T	2.75 × 10^-8^ D	1.33(1.20-1.47)	N
***C5orf56-IRF1***	rs4705862	T	0.42/0.45	0.11	0.87 (0.73-1.04)	0.033 D	1.02 × 10^-8^ A	0.84(0.79-0.89)	Y
*CCR1-CCR3*	rs79893749	T	0.09/0.11	0.10	0.78 (0.58-1.05)	0.10 A	1.88 × 10^-7^ A	0.78(0.72-0.86)	S
***ERAP2-LNPEP***	rs27290	G	0.47/0.39	0.00021	1.40 (1.17-1.66)	0.00021 A	7.50 × 10^-9^ D	1.32(1.20-1.45)	Y
***FAS***	rs7069750	G	0.47/0.45	0.19	1.12 (0.94-1.34)	0.19 A	2.93 × 10^-8^ A	1.18(1.11-1.25)	S
***IL2-IL21***	rs1479924	G	0.27/0.28	0.54	0.94 (0.77-1.15)	0.16 R	6.24 × 10^-11^ A	0.79(0.74-0.85)	S
***IL2RB***	rs2284033	A	0.40/0.43	0.24	0.90 (0.75-1.08)	0.18 D	1.55 × 10^-8^ A	0.84(0.79-0.89)	S
*IL6*	rs7808122	T	0.41/0.40	0.76	1.03 (0.86-1.23)	0.59 R	5.80 × 10^-8^ A	1.19(1.11-1.25)	N
*JAZF1*	rs10280937	C	0.15/0.14	0.77	1.04 (0.81-1.33)	0.74 D	6.60 × 10^-7^ A	1.25(1.15-1.37)	N
*LTBR*	rs2364480	C	0.26/0.27	0.65	0.96 (0.78-1.16)	0.49 R	5.10 × 10^-8^ A	1.20(1.12-1.28)	N
*PRR5L*	rs4755450	A	0.34/0.38	0.043	0.83 (0.69-0.99)	0.042 D	3.35 × 10^-7^ D	0.80(0.74-0.87)	Y
***RUNX1***	rs9979383	C	0.32/0.37	0.013	0.79 (0.66-0.95)	0.013 A	1.06 × 10^-8^ D	0.78(0.72-0.85)	Y
*RUNX3*	rs4648881	A	0.48/0.50	0.37	0.92 (0.78-1.10)	0.19 D	4.66 × 10^-7^ A	1.16(1.10-1.23)	S*
***TYK2***	rs34536443	C	0.02/0.03	0.10	0.63 (0.36-1.10)	0.10 A	1.00 × 10^-10^ A	0.56(0.47-0.67)	S
***UBE2L3***	rs2266959	T	0.23/0.19	0.040	1.25 (1.01-1.55)	0.036 T	6.20 × 10^-9^ D	1.24(1.15-1.33)	Y
***ZFP36L1***	rs12434551	T	0.46/0.45	0.61	1.05 (0.88-1.25)	0.50 R	1.59 × 10^-8^ D	0.77(0.71-0.85)	N

^a^Model providing most significant P value: A = allelic, D = dominant, G = genotypic, R = recessive, T = trend (Cochrane-Armitage Trend Test, also referred to as additive).

^b^Immunochip model: A = additive, D = dominant.

^c^Evidence for replication Y = yes N = no S = suggestive * = opposite direction of effect due to the minor allele being opposite between the two studies.

**Bold** = reached genome-wide significance in Immunochip paper.

**Figure 1:**
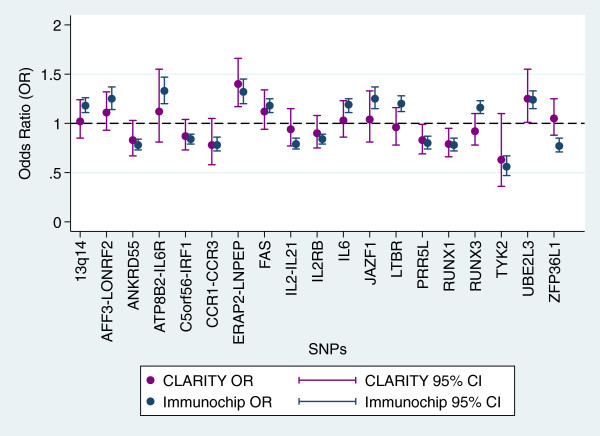
**Forest Plot comparing CLARITY allelic ORs with published Immunochip ORs for each SNP genotyped.**

Table 1 shows the ORs and p values for allelic association tests, as well as the ‘best test’ model and corresponding p value. These are shown alongside the reported ORs, p value, and model from the Immunochip study. Figure 1 shows a forest plot of the allelic odds ratios generated in the CLARITY sample compared to the Immunochip study ORs for all SNPs tested. We report replication of association for six genes (SNPs) in our sample - *AFF3-LONRF2* (rs6740838), *C5orf56-IRF1* (rs4705862), *ERAP2-LNPEP* (rs27290), *PRR5L* (rs4755450), *RUNX1* (rs9979383) and *UBE2L3* (rs2266959). A further seven genes (SNPs) showed suggestive association (defined as an OR in a consistent direction and a p < 0.2) - *ANKRD55* (rs71624119), *CCR1-CCR3* (rs79893749), *FAS* (rs7069750), *IL2-IL21* (rs1479924), *IL2RB* (rs2284033), *RUNX3* (rs4648881) and *TYK2* (rs34536442).

We re-analysed the data after restricting the samples to those of European descent to ensure associations were not being masked by differences in ethnicity between the prior study and our own. Only those individuals who identified all four grandparents as European were included. Individuals with a non-European grandparent or any missing information were excluded. This restriction criterion reduced our sample size to 229 cases (mean age 6.2 years, 70% female) and 418 controls (mean age 7.2 years, 40% female). The results of this analysis are detailed in Additional file 1: Table S2. Overall, the results of this analysis did not considerably differ from the entire sample, with a few exceptions. Of note, *RUNX3* (rs4648881), which had a suggestive association in the total sample, showed a significant association in the European-only sample. Furthermore, *ZFP36L1* (rs12434551) showed significant association with JIA in the European-only sample, suggesting it may be particularly sensitive to ethnicity. However, our reported SNP (T) differs to the previously reported SNP (A), and the association is in the opposite direction. As this SNP is an A/T transversion with frequencies of both alleles near 50% we cannot determine whether the direction of effect difference is due to allele reversal, and thus replicates the prior result, or the result of analysing different DNA strands between the two studies, in which case the risk locus is not replicated. Two genes that were significantly associated in the entire sample, *PRR5L* (rs4755450), and *AFF3-LONRF2* (rs6740838), were no longer significant in the European-only analysis, and *C5orf56-IRF1* (rs4705862) showed only suggestive significance. Given that the reduced sample size likely resulted in a reduction in statistical power, small movements in the ORs and p values are not unexpected, and this result is unlikely to indicate failure to replicate.

We have replicated the association of *AFF3-LONRF2* (rs6740838), and showed suggestive association of *ANKRD55* (rs71624119) and *IL2-IL21* (rs1479924), which all had evidence of association with JIA prior to the Immunochip study [11–13], although the samples used in those prior studies significantly overlapped with those used in the Immunochip study (i.e. the replication efforts were not independent). A recent independent study by Reinards et al. [14] also examined evidence for association of JIA with SNPs at *ANKRD55* and *AFF3* that are highly correlated (r^2^ ~ 0.9) with the Immunochip SNPs at these genes. The association with *ANKRD55* was replicated, further supporting a role for this gene in JIA risk. The association with *AFF3* was not replicated, although the direction of effect is consistent across studies.

Although our study has been able to confirm association of a number of Immunochip-identified genes with JIA risk, lack of replication in our study cannot be taken as evidence to refute association for a number of reasons. First, our sample size is much smaller than that used by the Immunochip study, and therefore has lower statistical power to detect association. This is somewhat offset by the more relaxed level of significance required for replication, compared to that required in the discovery study. Second, the Immunochip study was restricted to oligoarticular and polyarticular RF negative JIA. Here we have considered all JIA subtypes together. We chose not to perform subtype specific analyses, as the benefit of reduced heterogeneity would be offset by the reduction in statistical power associated with reduced sample size. The fact that the associations identified in this study were still detectable when all subtypes were considered together suggests common pathways to disease in all subtypes (potential ‘pan-JIA’ genes). Such genes are not unexpected, given the significant overlap amongst disease risk genes across far more clinically heterogeneous forms of autoimmune disease [15]. Despite the sample size and case heterogeneity limitations of our study, it is worth noting that a number of the associations that we have failed to replicate only carried a suggestive level of association in the original study, and therefore our lack of replication may well be indicating lack of true association.

This study has further confirmed association of JIA with seven genetic loci, six of which have now been replicated for the first time (*C5orf56-IRF1* (rs4705862), *ERAP2-LNPEP* (rs27290), *PRR5L* (rs4755450), *RUNX1* (rs9979383), *RUNX3* (rs4648881), and *UBE2L3* (rs2266959)). For an additional six genes, we generated weaker indication of association, further supporting (but not yet confirming) involvement in JIA risk. Further analysis of these loci in other independent samples will assist in clarifying their role in disease susceptibility. Overall, our study makes an important contribution to the growing body of international data that is revealing the genetic risk landscape of this paediatric autoimmune disease.

## Electronic supplementary material

Additional file 1: Table S1: Significant/suggestive SNPs from the Immunochip study not included in this replication analysis. Table S2: CLARITY SNP replication results in European---restricted cohort, case n=220 control n=418. (PDF 83 KB)
