# Supplementary material for: Independent confirmation of juvenile idiopathic arthritis genetic risk loci previously identified by immunochip array analysis
Source: Pediatr Rheumatol Online J. 2014 Dec 16;12:53. doi: 10.1186/1546-0096-12-53 (PMC4276094; doi:10.1186/1546-0096-12-53)
Supplement: Supplementary file 1 — Additional file 1: Table S1: Significant/suggestive SNPs from the Immunochip study not included in this replication analysis. Table S2: CLARITY SNP replication results in European---restricted cohort, case n=220 control n=418. (PDF 83 KB) [file 12969_2014_2174_MOESM1_ESM.pdf]

Supplementary Table 1: Significant/suggestive SNPs from the Immunochip study not included in this replication analysis

**Gene**	**SNP**	**Reason**[a]	**Previously published data for our sample**[b] **[1]**
HLA-DQB1-HLA-DQA2	rs7775055	Established risk factor for JIA	N/A
PTPN22	rs6679677	Established risk factor for JIA<br>In LD r^2=1 with rs2476601 which has previously reached GWS	rs2476601<br>OR 1.62 (95% CI 1.15-2.30) p=0.006
PTPN2	rs2847293	Established risk factor for JIA<br>In LD r^2=0.94 with rs1893217 which has previously reached GWS	rs1893217<br>OR 1.09 (95% CI 0.85-1.40) p=0.5
STAT4	rs10174238	In LD r^2=0.79 with rs7574865<br>Supported by previous studies, had not reached GWS prior to Immunochip	rs7574865<br>OR 1.29 (95% CI 1.03-1.61) p=0.025
COG6	rs7993214	Supported by previous studies	rs7993214<br>OR 1.09 (95% CI 0.88-1.36) p=0.41
TIMMDC1-CD80	rs4688013	In LD r^2=1 with rs4688011<br>Supported by previous studies	rs4688011<br>OR 1.32 (95% CI 1.04-1.68) p=0.02
IL2-RA	rs7909519	Failed assay design	
SH2B3-ATXN2	rs3184504	Failed assay design	
PRM1-RM12	rs66718203	Failed quality control	

[a] Linkage Disequilibrium estimated in CEU population using SNAP [2]

[b] Results from a smaller subset of the CLARITY cohort [1]

GWS= genome-wide significance

Supplementary Table 2: CLARITY SNP replication results in European-restricted cohort, case n=220 control n=418

Gene	SNP	Minor allele	Case MAF/ Control MAF	Allelic P	Allelic OR (95% CI)	Best P[a]	Immunochip P[b]	Immunochip OR(95%CI)	Replication[c]
13q14	rs34132030	T	0.30/0.31	0.61	0.94 (0.73-1.20)	0.28 R	$1.77x10^{-7}$ A	1.18(1.11-1.26)	N
AFF3-LONRF2	rs6740838	T	0.41/0.41	0.85	0.98 (0.78-1.23)	0.62 D	$8.83x10^{-7}$ D	1.25(1.14-1.37)	N
ANKRD55	rs71624119	A	0.21/0.24	0.19	0.83 (0.63-1.10)	0.19 A	$4.40x10^{-11}$ A	0.78 (0.73-0.84)	S
ATP8B2-IL6R	rs11265608	A	0.09/0.08	0.60	1.12 (0.74-1.68)	0.60 T	$2.75x10^{-8}$ D	1.33(1.20-1.47)	N
C5orf56-IRF1	rs4705862	T	0.39/0.43	0.17	0.85 (0.67-1.07)	0.13 D	$1.02x10^{-8}$ A	0.84(0.79-0.89)	S
CCR1-CCR3	rs79893749	T	0.10/0.13	0.08	0.72 (0.50-1.04)	0.076 A	$1.88x10^{-7}$ A	0.78(0.72-0.86)	S
ERAP2-LNPEP	rs27290	G	0.48/0.39	0.005	1.39 (1.11-1.75)	0.0045 T	$7.50x10^{-9}$ D	1.32(1.20-1.45)	Y
FAS	rs7069750	G	0.47/0.44	0.18	1.17 (0.93-1.47)	0.15 D	$2.93x10^{-8}$ A	1.18 (1.11-1.25)	S
IL2-IL21	rs1479924	G	0.25/0.29	0.17	0.83 (0.64-1.08)	0.16 T	$6.24x10^{-11}$ A	0.79(0.74-0.85)	S
IL2RB	rs2284033	A	0.40/0.43	0.17	0.85 (0.68-1.07)	0.13 D	$1.55x10^{-8}$ A	0.84(0.79-0.89)	S
IL6	rs7808122	T	0.48/0.45	0.46	1.09 (0.87-1.37)	0.23 R	$5.80x10^{-8}$ A	1.19(1.11-1.25)	N
JAZF1	rs10280937	C	0.12/0.14	0.57	0.91 (0.64-1.28)	0.57 A	$6.60x10^{-7}$ A	1.25(1.15-1.37)	N
LTBR	rs2364480	C	0.27/0.28	0.71	0.95 (0.74-1.23)	0.28 R	$5.10x10^{-8}$ A	1.20(1.12-1.28)	N
PRR5L	rs4755450	A	0.34/0.35	0.68	0.95 (0.75-1.21)	0.37 D	$3.35x10^{-7}$ D	0.80 (0.74-0.87)	N
RUNX1	rs9979383	C	0.30/0.37	0.01	0.71 (0.56-0.91)	0.006 T	$1.06x10^{-8}$ D	0.78(0.72-0.85)	Y
RUNX3	rs4648881	A	0.47/0.52	0.10	0.82 (0.66-1.04)	0.013 D	$4.66x10^{-7}$ A	1.16(1.10-1.23)	Y*
TYK2	rs34536443	C	0.02/0.04	0.07	0.53 (0.26-1.08)	0.074 A	$1.00x10^{-10}$ D	0.56(0.47-0.67)	S
UBE2L3	rs2266959	T	0.22/0.16	0.004	1.51 (1.13-2.01)	0.0036 T	$6.20x10^{-9}$ D	1.24(1.15-1.33)	Y
ZFP36L1	rs12434551	T	0.48/0.42	0.06	1.25 (0.99-1.57)	0.014 R	$1.59x10^{-8}$ D	0.77(0.71-0.85)	?

[a] Model providing most significant P value in best test association: A=allelic, D=dominant, G=genotypic, R=recessive, T= trend (Cochrane-Armitage Trend Test, also referred to as additive).

[b] Immunochip model: A=additive, D=dominant

[c] Evidence for replication Y=yes N=no S=suggestive *=opposite direction of effect due to the minor allele being opposite between the two studies

? Opposite direction of effect, unable to determine if strand difference or allele reversal

Bold = reached genome-wide significance in Immunochip paper

References

1. Ellis JA, Chavez RA, Pezic A, Ponsonby AL, Akikusa JD, Allen RC, Munro JE: **Independent replication analysis of genetic loci with previous evidence of association with juvenile idiopathic arthritis.** *Pediatr Rheumatol Online J* 2013, **11:**12.
2. Johnson AD, Handsaker RE, Pulit SL, Nizzari MM, O'Donnell CJ, de Bakker PI: **SNAP: a web-based tool for identification and annotation of proxy SNPs using HapMap.** *Bioinformatics* 2008, **24:**2938-2939.
